# Post-Stroke Neuropsychiatric Complications: Types, Pathogenesis, and Therapeutic Intervention

**DOI:** 10.14336/AD.2023.0310-2

**Published:** 2023-12-01

**Authors:** Jing Zhou, Yijia Fangma, Zhong Chen, Yanrong Zheng

**Affiliations:** Key Laboratory of Neuropharmacology and Translational Medicine of Zhejiang Province, School of Pharmaceutical Sciences, Zhejiang Chinese Medical University, Hangzhou, Zhejiang, China.

**Keywords:** Complications, neurological diseases, pathogenesis, post-stroke, psychiatric disorders

## Abstract

Almost all stroke survivors suffer physical disabilities and neuropsychiatric disturbances, which can be briefly divided into post-stroke neurological diseases and post-stroke psychiatric disorders. The former type mainly includes post-stroke pain, post-stroke epilepsy, and post-stroke dementia while the latter one includes post-stroke depression, post-stroke anxiety, post-stroke apathy and post-stroke fatigue. Multiple risk factors are related to these post-stroke neuropsychiatric complications, such as age, gender, lifestyle, stroke type, medication, lesion location, and comorbidities. Recent studies have revealed several critical mechanisms underlying these complications, namely inflammatory response, dysregulation of the hypothalamic pituitary adrenal axis, cholinergic dysfunction, reduced level of 5-hydroxytryptamine, glutamate-mediated excitotoxicity and mitochondrial dysfunction. Moreover, clinical efforts have successfully given birth to many practical pharmaceutic strategies, such as anti-inflammatory medications, acetylcholinesterase inhibitors, and selective serotonin reuptake inhibitors, as well as diverse rehabilitative modalities to help patients physically and mentally. However, the efficacy of these interventions is still under debate. Further investigations into these post-stroke neuropsychiatric complications, from both basic and clinical perspectives, are urgent for the development of effective treatment strategies.

## 1. Introduction

Stroke is one of the leading causes of death and disability worldwide, and has become a major economic burden owing to the costs of long-term rehabilitative care [1, 2]. With the rapid growth of the elderly population, the incidence of stroke is increasing at an alarming trend, increasing age remains the most threatening risk factor for patients suffering from stroke in both men and women [1]. Although improved drug treatment and advances in healthcare have reduced stroke mortality, poor outcomes are common in long-term survivors, accompanied by secondary complications, including cognitive impairment and dementia, pain, anxiety, depression, fatigue, and epilepsy [3, 4]. These complications substantially increase the risk of subsequent recurrence and later mortality. Here, we sought to review the available evidence to elucidate these different types of complications, and refine the definition, risk factors, prevalence, potential mechanisms, and therapeutic interventions, despite their overlap and interrelationship with each other. The mechanisms underlying post-stroke neuropsychiatric complications (PSNCs) are complex, involving multiple neurobiological cytokines and neurotransmitters systems [5]. Evidence has supported that inflammatory response, dysregulation of the hypothalamic pituitary adrenal (HPA) axis, cholinergic dysfunction, reduced level of 5-hydroxytryptamine (5-HT, also called serotonin), glutamate-mediated excitotoxicity, and mitochondrial dysfunction are involved in PSNCs, of which inflammation, dysfunction of the HPA axis and mitochondrial dysfunction affect almost all of the types of complications [6, 7]. While glutaminergic, cholinergic, and serotoninergic dysfunction have been suggested as key determinants of PSNCs, it must be noted that PSNCs, similar to other psychiatric disorders, are the consequence of abnormal brain functional network involving diverse neurotransmitter systems. Interestingly, as the most well-established neurotransmitter involved in mood disorders, dopamine has been found to play a role in the comorbidity of cardiovascular diseases with mood disorders, and thus the dopaminergic contribution to PSNCs is definitely worth further clarification [5, 7]. The prevention and treatment of post-stroke secondary symptoms remain challenging, partially attributable to multiple pre-stroke risk factors, diverse types, and characteristics of stroke [8, 9]. The discussion of these possible secondary complications provides physicians, nurses, and allied health professionals with comprehensive awareness of acute and long-term PSNCs, which enables them to provide stroke survivors with appropriate preventative care and intervention during acute care and rehabilitation [4].

## 2. Methods

In this narrative review, we searched stroke-related studies in the English language only, with no restrictions on research objective or publication type. The search terms in the “Therapy” section are restricted to human studies. Databases including PubMed, Medline, and Web of Science were searched for all relevant documents, mainly in the previous two decades, with the exception of several older representative studies. The search terms were as follows: ("stroke" OR "ischemia stroke" OR "hemorrhagic stroke") AND ("cognitive impairment" OR "cognition" OR "dementia" OR "memory impairment"). Additionally, we searched for publications on stroke therapy using the following search terms: ("stroke" OR "ischemia stroke" OR "hemorrhagic stroke") AND ("cognitive impairment" OR "cognition" OR "dementia" OR "memory impairment ") AND ("therapy" OR "intervention" OR "treatment"). The search terms for other PSNCs are similar to those listed above. The search terms used for the other PSNCs were as follows: "pain", or "epilepsy", "seizure", "epileptogenesis", "epilepsia", or "depression", "depressive", "depressed", or "anxiety", or "apathy", or "fatigue". Moreover, we reviewed the reference lists in retrieved publications.

**Figure 1.**
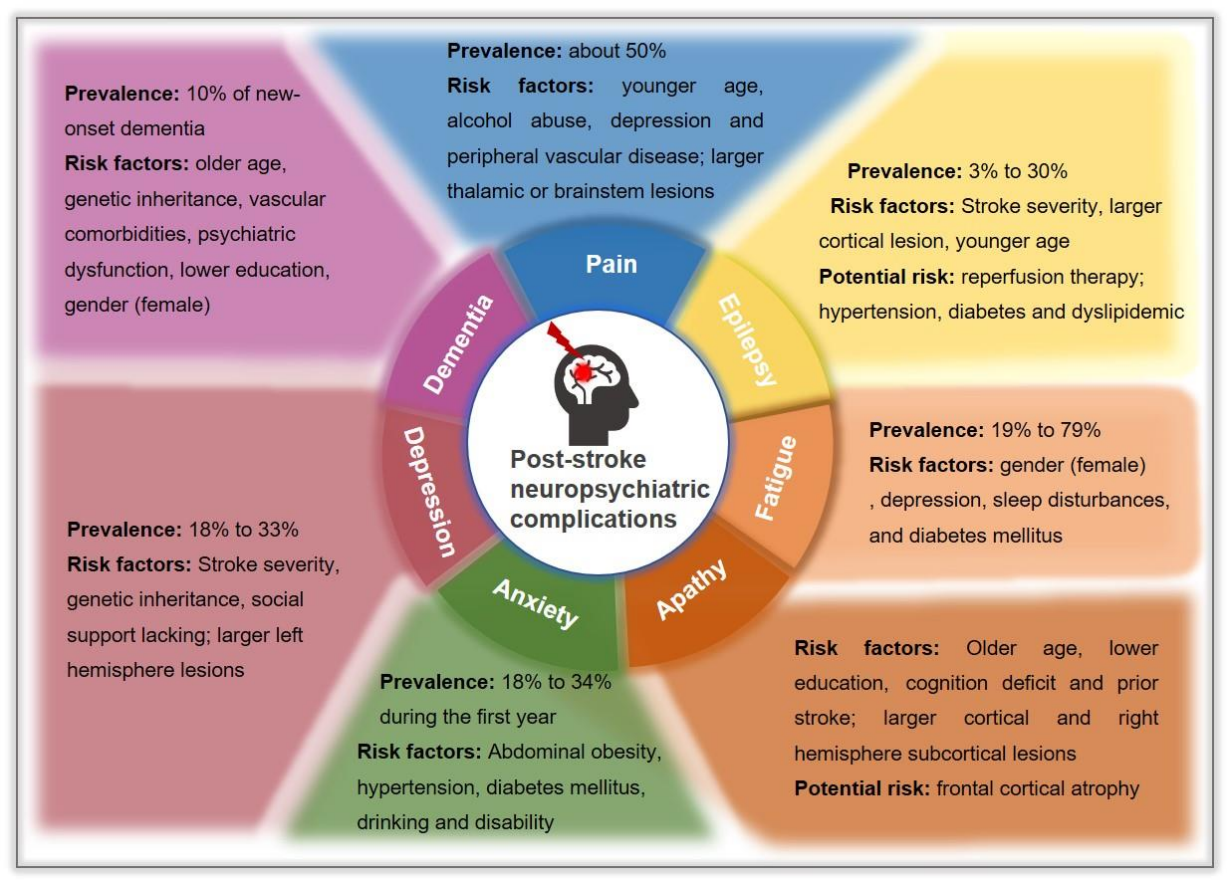
The prevalence, risk factors and potential risk of different post-stroke neuropsychiatric complications.

## 3. Classification and clinical features of post-stroke neuropsychiatric complications

We generally categorize PSNCs into neurological diseases, including pain, epilepsy, dementia, and psychiatric disorders, including depression, anxiety, apathy and fatigue. We will elaborate on the prevalence, occurrence characteristics, risk factors, vulnerability, and disease progression of different PCNCs. The risk factors of these different complications are summarized in Figure 1.

### 3.1. Neurological diseases

#### 3.1.1. Post-stroke cognitive impairment and dementia

Previous reports confirmed that long-term functional deficits occur in most stroke survivors, causing chronic disability. Post-stroke cognitive impairment and dementia (PSCID) are fairly common in stroke survivors. Generally, dementia is defined as moderate-to-severe cognitive impairment that occurs within 3 months after incident stroke. Cognitive impairment often occurs as both a predictor and consequence of stroke incidence [10]. According to extensive data, over 50% of stroke survivors develop varying degrees of cognitive impairment, and approximately 25% develop dementia [10, 11]. To be more precise, reliable data have refered that the incidence of developing new-onset dementia after the initial stroke is 10%, and more than one-third of patients develop dementia after recurrent stroke [12]. Otherwise, a study aimed at US adults showed that women were more likely to develop post-stroke dementia than men, especially during the early period after stroke onset [13]. Moreover, it was suggested that the impact of acute stroke on cognition showed no racial differences in older individuals [14].

Risk factors for PSCID are multifactorial, including increasing age, genetic inheritance, undereducated status, socioeconomic disparity, vascular comorbidities, and psychiatric dysfunction. It has been well documented that older age is the leading risk factor for PSCID, which is consistent with the high morbidity of stroke-related dementia in elderly [15]. Most notably, elderly individuals who have any comorbidity with vascular risk factors, such as diabetes and atrial fibrillation, are at higher risk of PSCID [16]. Interestingly, hypertension, high cholesterol, smoking, and alcohol use, which strongly contribute to stroke, have no direct effect on increasing the risk of PSCID, yet the intervention focused on these factors still preserves cognition decline through preventing recurrent stroke [16, 17]. Additionally, studies have found that longer educational history gives rise to less PSCID, which brings about a favorable long-term survival [12, 13].

Currently, it is widely accepted that PSCID is associated with psychiatric disorders. Available data have suggested that experiencing delirium in the acute stage of stroke predicted more cognitive impairment, and PSCID often co-exists with mood disorders and apathy[18]. Also, it is well-known that dementia is closely associated with memory decrements, research has confirmed that stroke onset triggers memory impairment, and those who suffered stroke experienced faster memory decline in the years prior to stroke compared with stroke-free populations [19]. Although PSCID occurs frequently, it is often overlooked when it coincides with other worrisome symptoms. Thus, it is critically important to specifically assess for PSCID during follow-up care, which may reduce the risk of permanent institutionalization and prolong survival [11].

#### 3.1.2. Post-stroke pain

Pain almost runs through all stages of stroke. Indeed, post-stroke pain (PSP) affects up to 50% of stroke survivors [20]. Most clinical management after stroke focuses on reducing the risk of recurrence and recovering motor and neurologic functions, as well as treating aphasia, dementia or neglect syndrome, causing a direct consequence of underdiagnosis and undertreatment of PSP [21]. The prevalence and annual incidence of PSP is difficult to define accurately given the different types, intensities, and post-stroke phases and follow-up periods. Some research shows that almost half of patients suffer various degrees and types of pain in different period, and the highest incidence of PSP occurs in the subacute phase after stroke [20-22]. Common PSP subtypes include central post-stroke pain (CPSP), regional pain syndromes, joint pain, spasticity-related pain, headache, and musculoskeletal pain, such as the most common hemiplegic shoulder pain, all of which negatively affect daily functional abilities of stroke survivors [23].

Among the various types of PSP, CPSP is the most common and unbearable pain. CPSP is a dynamic condition that occurs as constant or intermittent neuropathic pain symptoms accompanied by sensory hyposensitivity, such as temperature dysesthesia [24]. Since pain caused by ischemic injury to the central nervous system (CNS) is difficult or vague to characterize or classify, patients may describe painful sensations that are indefinitely positioned or that vary over time. As a consequence of ischemia stroke, headache emergence can also be considered as a precursor of stroke, and several studies have demonstrated that headache occurs mainly in the acute stage after stroke, and that a primary headache disorder doubles the risk of ischemic stroke [25]. While hemiplegic shoulder pain is a typically delayed complication that usually occurs 2-3 months after stroke, it is often negligible in the acute phase and then markedly amplified in the subacute phase [26].

Older age and female gender are well-recognized demographic characteristics related to any PSP syndrome [20, 25]. However, it is interesting to note that younger patients are at higher risk for CPSP [27]. Pre-stroke features, such as alcohol abuse, statin use, depression and peripheral vascular disease, are identified as independent risk factors of aggravating PSP [28]. Clinically, PSP is always accompanied by symptoms of increased muscle tone, sensory deficits, and upper limb motor paralysis. Additionally, stroke site and mechanism, including thalamic and brainstem localization, also play an essential role in the initiation and development of PSP [23]. Accordingly, follow-up records have shown that pain can lead to a series of comorbidities, such as depression[29], fatigue [30], sleep disturbances [31], and in severe cases, even suicidal tendencies. With the aim to ameliorate long-term outcomes and quality of life of stroke survivors, clinicians’ active acquirement and patients’ spontaneous complaints of pain sensation will contribute to earlier identification and intervention of PSP.

#### 3.1.3. Post-stroke epilepsy

Stroke is the leading cause of epilepsy in older adults, accounting for nearly half of all epilepsy cases, and post-stroke epilepsy (PSE) is closely related to increased morbidity and mortality [32]. Over the last decades, progress in post-stroke healthcare and management has allowed more patients to survive, correspondingly, though, the occurrence of PSE is also observed raised. The International League Against Epilepsy (ILAE) defines early or acute symptomatic seizures (ASSs) as those that occur within 7 days after stroke, whereas remote symptomatic seizures are those that occur after 7 days [33]. Generally, only late post-stroke seizures are considered as PSE because ASSs have a low relapse rate, whereas late-onset seizures have a high relapse rate of approximately 70%, which is essential basis for the diagnosis of PSE [34]. In addition, patients with ASSs occurring between 4-7 days after stroke-onset were at higher risk of developing PSE [32].

The cumulative incidence rate of PSE ranges from 3% to 30%, and no significant difference in incidence rate has been observed between men and women [34-36]. Patients who suffer a hemorrhagic stroke are more likely to develop epilepsy than patients who have an ischemic stroke, occurring in approximately 6% and 10%, respectively [35, 37]. Currently, generally accepted risk factors of PSE include stroke severity, degree of neurological damage, large lesion volumes (especially in cortex), younger age at stroke onset, and hemorrhage [38]. It is noteworthy that considerable proportion of younger, even neonate, suffer ischemic stroke, who has a greater propensity for epileptogenesis and long-term unfavorable functional outcomes [39]. Finally, two studies have reported that the ALDH2 rs671and T allele of the D40-1C/T polymorphism are associated with PSE susceptibility [40, 41].

The association between reperfusion therapy and risk of post-stroke epileptic seizures presents conflict. An earlier study suggested that seizures might be a marker of successful reperfusion [42]. A meta-analysis concluded that there is insufficient evidence to prove that reperfusion therapy, either using intravenous thrombolysis with recombinant tissue-type plasminogen activator (rt-PA) or mechanical thrombectomy (MT), directly increased risk of seizures [35], although it may augment the risk of seizures by causing hemorrhagic transformation (HT) [43]. Otherwise, some studies held that patients undergoing reperfusion therapy with intravenous rt-PA or intra-arterial therapies were susceptible to developing PSE, with a phenomenon of lowering seizure threshold, and early data from several animal studies have also supported this finding [44, 45]. Of note, the aforementioned clinical studies achieved consensus that combined therapy of rt-PA and MT had no additive negative effect on the occurrence of seizures. The contribution of reperfusion therapy to acquired PSE awaits further investigation. Moreover, whether stroke-related diseases, such as hypertension, diabetes, and dyslipidemia, are responsible for PSE has not been definitively concluded. Several studies have reported that depression and dementia are involved in PSE, and that patients with depression or impaired cognition who encountered a stroke had an increased likelihood of experiencing PSE seizures [34, 45].

### 3.2. Psychiatric disorders

Considering the often-devastating impact of stroke on physical function, clinicians can overlook the impact of stroke on the mental health of patients, thus normalizing psychiatric disorders, and causing delayed (or even non-existent) diagnosis and treatment.

### 3.2.1. Post-stroke depression

Depression is the most frequent post-stroke psychiatric disorder, with an estimated prevalence range from 18% to 33% [46, 47]. Patients who suffer post-stroke depression (PSD) are more vulnerable to cognitive deficits, suicidal tendencies and long-term disability, ultimately resulting in poor quality of life and higher mortality. Recent reports have shown that the incidence rate of PSD is higher within the first year after stroke onset, which is reported by approximately 33% and then decreases to about 25% after 1 year [46, 47]. An accumulating body of evidence focused on the analyzing risk factors of PSD has emerged to help clinicians identify and prevent PSD symptoms. Several lines of research have indicated that females are more susceptible to depression after stroke, yet are less likely to receive treatment than males [46, 48]. However, some studies considered no connection between gender and PSD, thus, whether incidence of PSD is differentiated by gender remains controversial [49]. Stroke severity including various lesion type, volume, location and laterality, is judged to be the most robust facilitator for PSD[50]. It is generally recognized that patients with large infarct volumes, left hemisphere lesions, and infarct in the anterior/frontal areas and basal ganglia have a higher likelihood of developing PSD [46]. In addition, no difference in the incidence and mechanism of PSD has been found between hemorrhagic stroke and ischemic stroke. Furthermore, both individual and familial history of psychiatric diseases, and lack of social support during stroke may aggravate the possibility of PSD [51].

### 3.2.2. Post-stroke apathy

Patients with depression who suffer a stroke frequently suffer from apathy, a common neuropsychiatric symptom that occurs in about one-third of stroke patients. Apathy refers to the simultaneous decrease of goal-oriented activities in the cognitive, behavioral, emotional, or social fields of a patient's life due to diminished motivation [52]. Post-stroke apathy (PSAp) is often misdiagnosed as depression partially attributed to overlapping symptoms with depression, and both manifesting as an inactive mood. Although sharing some symptomatic characteristics, PSAp and depression differ in prevalence, pathogenesis, comorbidities, and outcomes [53]. Emotional distress is the key point to distinguish between apathy and depression: patients with PSD usually accompanied by pessimism and hopeless emotion, which rarely occur in patients with PSAp, who, conversely, often present with indifference and passive engagement with daily behaviors. Furthermore, neuroimaging findings have indicated that PSAp lesions are mainly distributed in the right hemispheric subcortical region, whereas PSD is linked to left anterior lesions [53]. More detailed, greater basal ganglia engage in initiation apathy, prefrontal cortex engages in executive apathy, and orbitofrontal cortex engages in emotional apathy [54]. Crucially, substantial reductions in white matter integrity were observed in patients with apathy [55]. It is of utmost importance for clinicians and caregivers to distinguish PSAp from depression to ensure patients receive appropriate treatment and effective subsequent management.

Intriguingly, except for well-established risk factors such as older age, lower education, and cognition deficit, PSAp was found to be independent of stroke severity or prior stroke [56]. Nevertheless, it has been found that frontal cortical atrophy is a radiological predictor of PSAp[57]. On the other hand, recent studies presented idea that apathy, instead of depression, might be a prodromal symptom of dementia in stroke, which provides some guidance for recognizing patients at-risk of mild cognitive impairment [52]. Considerable evidence has demonstrated that PSAp is associated with other adverse consequences of stroke, including functional impairment, delirium, and fatigue, and particularly with difficulty in concentrating and reduced information processing speed [58].

### 3.2.3. Post-stroke anxiety

Post-stroke anxiety (PSAn), a consequence of poor motor functioning and substantial decline in quality of life, is a common mental disorder that compromises patient rehabilitation, and, in turn, results in a lasting and steady deterioration of life quality. Previous accumulative studies have reported that PSAn occurs in 18% to 34% of survivors during the first year after stroke, and the rates did not lower meaningfully up to 5 years after stroke [59-62]. Nevertheless, as a serious psychological and physiological problem, PSAn is relatively neglected, presently, compared to other post-stroke psychological disorders. PSAn typically manifests as excessive concern on personal prognosis including recurrence of stroke, returning to work, falling, and ability to maintain independence, which bare considered normal responses to stroke, and are considerable challenges for the accurate diagnosis of PSAn [59]. An analytical study reported that patients who had a hemorrhagic stroke were at higher risk of developing PSAn and that hemorrhagic stroke was the main predictor of anxiety in the first year after stroke[61], yet no connection was found between lesion location or lesion side and PSAn [59]. Accordingly, the authors invoked that clinicians pay close attention to anxiety symptoms in patients with hemorrhagic stroke. Similar to PSD, reports in terms of associations between gender and PSAn are inconsistent. Some studies have deemed that female stroke survivors are more likely to develop PSAn, possibly due to female patients being more vulnerable to social pressure and other psychological factors, yet other researchers who disagreed considered no statistical link between gender and PSAn, or to the convert, men are more likely to experience anxiety after a stroke than women [59, 60, 62].

PSAn usually coexists with other threatening events such as cognitive impairment, depression, and insomnia, forming a chronic interaction in stroke patients. PSAn can exacerbate depression and cognitive impairment, and degrade patient prognosis, which possibly attributes to reduced social engagement and increased functional dependence in daily activities [63]. Most studies involving the relationship between age and PSAn conclude that age is not a risk factor of PSAn [59]. A preliminary study of obesity and PSAn proposed that abdominal obesity was independently related to PSAn but not to PSD, which was supported by the study of DeJesus et al. [64]. In addition, an analysis using machine learning models found that diabetes mellitus, hypertension, alcohol use, and disability increase the risk of PSAn, and that higher serum high-density lipoprotein cholesterol (HDL-C) level can decrease the risk of PSAn [59]. It has also been reported that vitamin D deficiency and serum levels of oxidative markers such as MDA, SOD, and CAT at admission for stroke can be predictors of PSAn at 1 month after the event. Cumulatively, these correlations could provide a guide in identifying and diagnosing PSAn in clinical practice.

### 3.2.4. Post-stroke fatigue

Fatigue is one of the most common and persistent consequences after stroke. Given substantial heterogeneity exists among various studies, the prevalence of post-stroke fatigue (PSF) has been roughly estimated at 19% to 79% [65-67]. Until recently, PSF was still a neglected issue since it is difficult to quantify and measure. Also, it is often characterized by early exhaustion, subjective lack of energy, and chronically existence that causes burnout towards exercise, emotion and cognition, and usually cannot be ameliorated by rest [67]. It also negatively affects functional outcomes in stroke survivors, leading to worse quality of life, less social participation, lower likelihood to return to work and higher mortality [68]. In line with this, PSF not only affects psychological disorders, such as depression and insomnia, but also causes sarcopenia [69]. PSF is affected by multiple risk factors, including age, gender, depression, lesion location, sleep disturbances, and diabetes mellitus [70]. On the flip side, chronic PSF appears to largely promote multiple comorbidities, such as depression, and it can be a crucial feature of depression. Depression and fatigue can be mutual predictors, the relationship between PSD and PSF does not necessarily mean that one factor will induce the other, nor does it rule out the possibility that a third factor leads to both [71]. Previous analyses reported that depression and anxiety can accelerate fatigue within half a year after stroke, and sometimes they were considered as consequences of fatigue rather than causative factors [63, 68]. Age is the most widely studied factor, however, conclusions regarding these findings are still controversial. Relatively more evidence supported that patients with older age are more likely to suffer PSF [72], whereas other studies shown that younger stroke survivors had a higher incidence of PSF [73, 74]. Moreover, a recent study conducted in Asian and European populations showed that female increased susceptibility to PSF, which is in parallel with the data that female predicts long-term PSF [65, 70]. In addition, there is no definite evidence indicates that cardiovascular disease is responsible for PSF [65, 70]. Thus far, data regarding whether lesion location is associated with PSF are inconclusive, although basal ganglia, brainstem, or thalamus lesions were more observed in stroke survivors with PSF [67, 71, 75]. Also, a higher proportion of patients with recurrent stroke had PSF compared to patients after a first stroke [76]. Considering the serious burden of PSF and its negative impact on patient survival, a better understanding of its etiology and pathogenesis is meaningful to develop new prevention strategies and treatments.

## 4. Mechanisms underlying post-stroke complications

**Figure 2.**
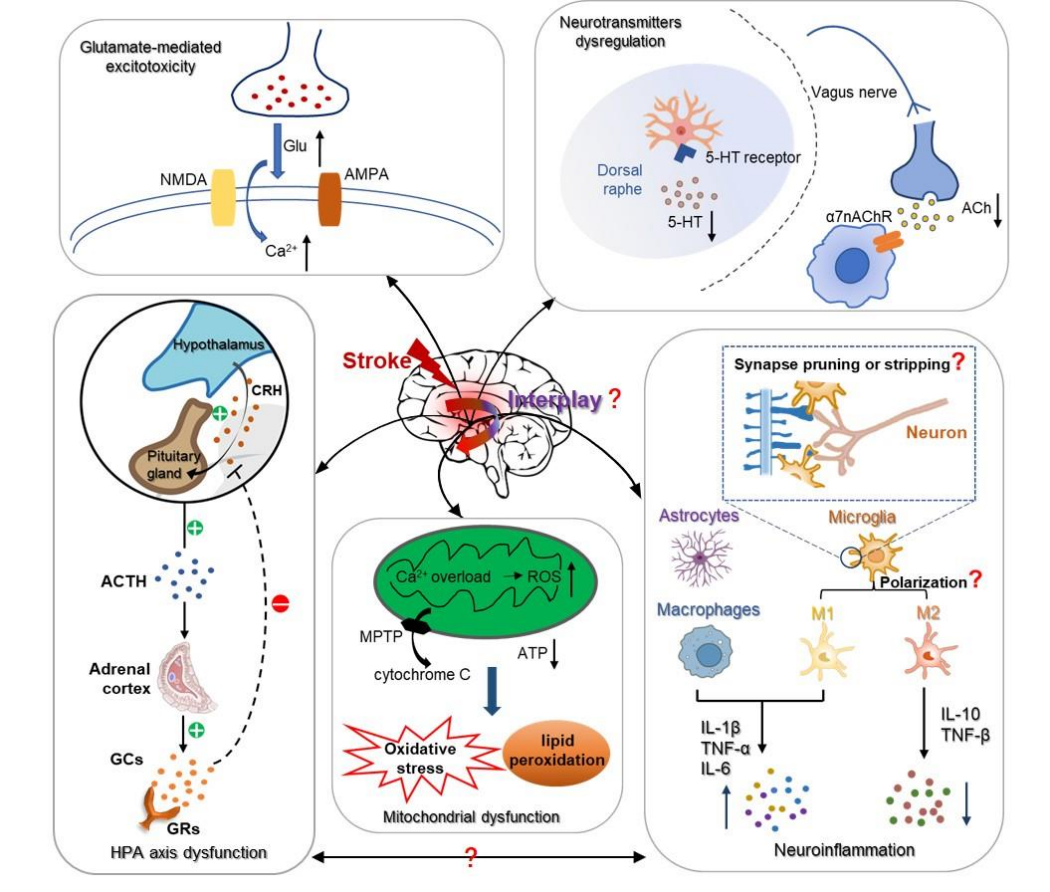
Schematic overview of mechanisms underlying post-stroke neuropsychiatric complications. In the acute phase, stroke as a stressor initiates the activation of the hypothalamic-pituitary-adrenal (HPA) axis and simultaneously stimulates the secretion of pro-inflammatory cytokines from immune cells, e.g., TNF-α, IL-1 and IL-6. The burst of systematic inflammation after stroke attenuates the inhibitory feedback of glucocorticoids (GCs) on hypothalamus and thus prolongs HPA axis activation. Moreover, stroke cascades affect diverse neurotransmitter pathways resulting in the reduced level of 5-HT and cholinergic dysfunction. Stroke also leads to the impaired release and reuptake of glutamate, as well as the overload of intracellular Ca^2+^ that promotes the rapid rise of glutamate levels in the cerebrospinal fluid. The activation of NMDA and AMPA GluR further induces glutamate toxicity. Excess ROS and Ca^2+^ accumulation induce mitochondrial dysfunction that leads to oxidative stress and lipid peroxidation. The MPTP open, followed by cytochrome C releasing from mitochondria into the cytoplasm. These dysregulations contribute to PSNCs, e.g., cognitive impairment, pain, epilepsy, depression, apathy and anxiety. However, the key brain areas responsible for serotoninergic and cholinergic dysfunction are still murky. Moreover, although microglia-mediate neuroinflammation has long been believed to play an essential role in stroke, the changes of microglia (like polarization, synapse pruning and synapse stripping) have not been dynamically observed in vivo in the context of PSNCs. Furthermore, the interplay between these pathological events awaits further study.

Interestingly, although the symptoms of PSNCs vary, the studies to date seem to support some general mechanisms underlying these complications, including inflammatory response, dysregulation of the HPA axis, cholinergic dysfunction, reduced level of 5-hydroxytryptamine, glutamate-mediated excitotoxicity, and mitochondrial dysfunction (Fig. 2).

### 4.1. Neuroinflammation

Several meta-analyses or longitudinal analyses have described findings of pro-inflammatory markers in patients with PSCID, PSD or PSP, and conversely, the increments of inflammatory markers can be predictors of these complications [77-79]. Additionally, both human and animal studies indicate that oxidative stress induced by inflammatory response is involved in neuronal death and seizures [80]. Patients with depression present higher blood concentrations of pro-inflammatory cytokines, including tumor necrosis factor-α (TNF-α), interleukin 1β (IL-1β), IL-6, and other proteins such as C-reactive protein during the acute phase. In turn, a stroke-induced immune reaction in the brain can also influence the progression of PSD [81]. Long-term neuroinflammation can strongly induce gray-matter and white-matter damage, resulting in long-term sensorimotor and cognitive impairment [82]. Pain can be driven by neuroinflammation via central sensitization in periphery and the CNS [79]. These results establish that inflammation and immune responses, as pivotal biological events, are highly relevant to stroke rehabilitation and neuropsychiatric complications [83].

The inflammatory processes can be further divided into peripheral and central inflammation. Microglia and astrocytes, as the main immunocompetent cells, engage in neuroinflammatory processes [84]. Abnormal neuroplasticity can be observed in several PSNCs, including depression and anxiety, pain, and cognitive impairment [16, 78, 85]. Microglia regulate neuroplasticity in various ways. After ischemic brain injury, microglia can be activated within minutes, and they can be recruited to the lesion sites and rapidly respond to injury signals by releasing pro-inflammatory cytokines [86]. Released cytokines, such as IL-1β and TNF-α, may account for hyperexcitability in neural networks underlying PSE [87]. Moreover, microglial activation has also been shown to suppress hippocampal neurogenesis, which aggravates cognitive dysfunction after stroke [88]. Furthermore, microglia may modulate synaptic plasticity directly by pruning or stripping synapses, both of which are worthy of further investigation in the context of PSNCs [89]. Nevertheless, there are contradictory findings supporting the beneficial effects of microglia in PSNCs. Activated microglia can attenuate neuronal apoptosis and enhance neurogenesis through engulfing cellular debris, and by secreting IL-10 and TGF-β cytokines [6]. More detailed, microglia can differentiate into two phenotypes—the detrimental pro-inflammatory M1 type and the protective and anti-inflammatory M2 type—which represents the dual role of microglia (Figure 2). The M1 type microglia can promote the release of a range of pro-inflammatory cytokines, such as TNF-α, IL-1β, IL-6, and nitric oxide (NO) as well as proteolytic enzymes, such as matrix metalloproteinase-9 (MMP-9) and MMP-2, which eventually aggravate neuronal injury and inhibit neurogenesis in the hippocampus [90]. The M2 type microglia can express protective cytokines such as CD206, TGF-β, and IL-10 as well as scavenge receptors, which is instrumental in inhibiting inflammation and promoting tissue repair. Within 7 days after stroke, microglia mainly polarize into the M1 proinflammatory phenotype, whereas the M2 anti-inflammatory phenotype subsequently predominates during the following 2 weeks [90, 91]. The polarization of microglia may explain the contradictory findings above; however, direct evidence is lacking.

In addition to microglia, astrocytes are also activated and proliferated following stroke, with the main characteristic of hypertrophy and ion channel remodeling, which may lead to secondary neurological disease such as epilepsy [80]. In the early phase of stroke, astrocytes can produce excess inflammatory cytokines, such as IL-1β or release glutamate and MMP-2 that causes degradation of matrix protein and subsequent blood-brain barrier (BBB) disruption, which eventually damages neuronal viability and causes encephaledema [92]. Furthermore, astrogliosis is also involved in the formation of glial scar that compromises neuronal regeneration, which is closely associated with neuroinflammation [90]. During stroke rehabilitation, astrocytes can also exert protective effects by uptaking glutamate and releasing neurotrophins to promote angiogenesis and neuronal plasticity [92, 93].

Accumulating evidence has demonstrated that CNS communicates with periphery through various mechanisms. Stress in stroke stimulates peripheral immune cells to secrete pro-inflammatory cytokines, TNF-α, IL-1β and IL-6, which can cross the BBB and further activate microglia and astrocytes in the CNS to secrete more pro-inflammatory cytokines. Ultimately, this causes widespread neuroinflammation and produces neurodegenerative disorders, such as dementia and epilepsy [84, 94]. Macrophages, as the main cells of the peripheral immune system, have been proved to participate in the prognosis of stroke via secreting pro-inflammatory cytokines, such as TNF-α and IL-6, and anti-inflammatory cytokines, such as IL-10 [83]. Similar to central inflammation, peripheral inflammation also has a biphasic effect, and the specific role of peripheral inflammation in determining outcomes after stroke requires further study.

### 4.2. Dysregulation of the HPA axis

The HPA axis is an important parcel of the neuroendocrine system and is involved in controlling stress responses and regulating many physiological activities, such as metabolic, cardiovascular, immune, and behavioral processes [85]. The connection between increased HPA axis activity and prognosis after ischemic stroke has long been confirmed. Olsson et al. reported that HPA axis hyperactivation has several negative effects on organism function activity and may be a predictor of a poorer functional prognosis after acute ischemic stroke [95]. Also, HPA axis hyperactivation may be critical in shaping pathophysiological reactions to ischemia. It has been well established that a series of psychiatric disorders, including panic, depression, phobias and anxiety, are affected by hyperactivation of the HPA axis [96]. Moreover, the HPA axis passively affects the structure and function of the temporal lobe of the brain, which is widely involved in epilepsy [97]. Stroke-induced seizures damage temporal lobe structures, which can further dis-restrain the HPA axis, trigger a vicious circle of neuronal injury, and predispose to subsequent seizures and psychiatric disorder comorbidities [97, 98].

In the acute phase, stroke as a stressor initiates a hyperactive sequence of the HPA axis: hypophysiotropic neurons secrete more corticotropin-releasing hormone (CRH), the pituitary releases more adrenocorticotropic hormone (ACTH), and then ACTH induces the adrenal cortex to synthesize and secrete more glucocorticoids (GCs), including cortisol (Fig. 2) [98]. Both clinical data and results from a study in an ischemia model show that GCs participate in stroke induced by brain dysfunction, indicating that GCs could potentially aggravate brain injury via activation of GC receptors and increase the vulnerability of neurons [96]. Many studies have shown that activation of these hormones after ischemic stroke could directly lead to neurohormonal dysfunction and neuronal and cell damage, which culminate in poor long-term prognosis [99]. Furthermore, abnormal responses with GCs over-release are regulated by low-affinity glucocorticoid receptors (GRs), which cause reduced inhibitory feedback (Fig. 2). Subsequently, GCs over-release brings about saturation of high-affinity mineralocorticoid receptors that are mainly distributed in the septa and hippocampus, and are responsible for the fundamental secretion of GCs, thereby augmenting neurohormonal dysfunction [100].

In addition to the positive and negative feedback mechanisms, modulation of the HPA axis can affect prognosis after stroke through GC-independent pathways, such as the neurotransmitter pathway. It was reported that, in acute ischemic stroke patients, elevated cortisol in plasma was negatively correlated with brain-derived neurotrophic factor (BDNF) levels, cognition, neurological status, functional responses, and emotional conditions, prompting a link between the decrease of clinical, behavioral, and blood biochemical parameters as well as stress-induced cortisol increase [101, 102]. Additionally, concentration of GCs resulted by HPA axis dysfunction impairs hippocampal neurogenesis, and reduced levels of BDNF in the hippocampus have also been observed [101]. Many studies have reported that patients with depression had lower BDNF levels in blood and hippocampus [102]. Stroke cascades involve hyper-glutamatergic transmission associated with excessive glutamate release, and the excitotoxicity caused by glutamate is reported to be of great importance in several neurological and psychiatric disorders [96]. Some researchers have proposed that glutamate might act on several brain regions, including hypothalamus, hippocampus and temporal cortex, and that glutamate may directly activate the HPA axis, which sequentially affect the secretion of hormones, such as CRH, ACTH, and GCs [103]. Abundant GRs in the hippocampus are essential for the physiological control of executive functions and behavioral responses to stressogenic factors. These effects accelerate post-stroke hippocampal degeneration and culminate in cognitive impairment. Moreover, it is well documented that the functioning of the HPA system is also related to neuroinflammation, and that progressive neuroinflammation caused by HPA axis dysfunction stimulates the expression of pro-inflammatory cytokines, such as TNF-α, IL-1β, and IL-6, which could further activate the HPA axis and cause increased levels of corticosterone [103]. In brief, inflammation, neurotransmitters and the HPA axis influence each other after stroke, and elicit a wide array of complications.

### 4.3. Cholinergic dysfunction

It is widely known that the central cholinergic system plays a pivotal role in learning and memory functions, but it is also involved in various pathological processes, such as epilepsy, depression, and anxiety [53, 104]. The broad effects of the cholinergic system may be due to its potent regulatory role in inflammatory immune responses [105]. There is substantial evidence that central cholinergic dysfunction is involved in vascular dementia pathogenesis [8]. Previous studies investigating the relationship between stroke and cholinergic activity have demonstrated a cholinergic deficit in stroke, indicated by a reduction of cholinergic markers. It has further been demonstrated that, the activity of acetylcholine esterase (AChE) dimers decreases with age, which provides additional evidence that aging patients are more likely to suffer post-stroke dementia [106]. At the onset of stroke, ischemia rapidly triggers a cascade of reactions, including the inflammatory response, oxidative stress, and apoptosis, which can lead to central cholinergic dysfunction in selectively vulnerable regions such as hippocampus and cerebral cortex, thereby exacerbating neuronal injury and cognitive decline [107]. Experimental evidence suggests that cerebral ischemia/reperfusion injury (CI/RI) or hypoperfusion also causes many sequelae that include loss of hippocampal neurons and behavioral deficits [8, 107]. Acetylcholine (ACh) is an important brain-body signaling route, the damaged tissue can secrete inflammatory cytokines to activate afferent signals through ascending vagus nerve fibers. In addition, there is a “cholinergic anti-inflammatory pathway” in which ACh interacts with α7 subunit-containing nicotinic receptors (α7nAChR) in immunocytes and promptly suppresses the release/synthesis of TNF-α and other inflammatory mediators, then preventing tissue damage [108]. Also, it was reported that vagus nerve stimulation has a neuroprotective effect in suppressing inflammation and apoptosis via activation of cholinergic pathways in acute CI/RI [109].

Reduced ACh levels and AChE activity have been observed in the brains of patients with cognitive impairment. Correspondingly, the use of cholinomimetic agents, such as cholinesterase inhibitors and ACh receptor agonist, has been an effective strategy for PSCID, and these drugs are also assumed to be a therapeutic strategy in the treatment of acute cerebral damage [108]. However, cholinomimetic agents have not consistently shown efficacy in cognitive impairment, and it is possible that they are only beneficial for individuals with an established cholinergic deficit [110]. Additionally, the clinical use of AChE inhibitors may reduce the risk of ischemic stroke and death in patients with dementia [111]. In summary, activating the cholinergic anti-inflammatory pathway may be a potential strategy for post-stroke therapy. However, detailed characterization of the molecular mechanism of ACh in stroke is lacking and requires further investigation.

### 4.4. Lower levels of 5-hydroxytryptamine

Presently, antidepressants, such as selective serotonin reuptake inhibitors (SSRIs) are the main therapeutic prescribed for post-stroke depression and apathy, as well as for pain and cognitive impairment, which underscores the importance of 5-HT in the pathogenesis of PSNCs [112, 113]. Serotoninergic neurons reside mainly in the dorsal raphe nucleus and can project to the entire brain and neuraxis. Previous studies have shown substantial upregulation of serotonergic receptors after the onset of stroke [9]. The injured projection could cause diminished levels of 5-HT in the left frontal cortex, resulting in depressed mood and cognitive impairment [46]. Likewise, neuroinflammation is involved in 5-HT metabolism. BDNF interacts with 5-HT to regulate neuronal plasticity, and exogenous BDNF increases the level of 5-HT[9]. Furthermore, there is evidence that 5-HT can mitigate glutamate efflux induced by ischemic human cerebrocortical slices [46]. Also, the HPA axis and 5-HT influence each other: HPA axis dysfunction followed by stroke affects the release of 5-HT, and decreased 5-HT further activates the HPA axis, which is additionally evidenced by higher cortisol levels in patients with depression [114].

### 4.5. Glutamate-mediated excitotoxicity

Glutamate is the major excitatory neurotransmitter in the CNS. Numerous studies have shown that excitotoxicity caused by pathological excessive release of glutamate is involved in stroke [115]. Following on, dysfunction of the glutamate transmission system is closely associated with the pathogenesis of neurodegenerative and neuro-psychiatric diseases after stroke, including depression, epilepsy, and cognitive impairment [116]. After the onset of stroke, brain tissue becomes acutely ischemic/hypoxic, resulting in ion transporter dysfunction and ion homeostasis disruption, which, in turn, lead to the impaired release and reuptake of glutamate, and overload of intracellular Ca^2+^ that further promotes the rapid rise of glutamate levels in the cerebrospinal fluid (CSF). These cascades ultimately lead to neuronal death [117, 118]. Moreover, the excessive release of glutamate could cause synaptic excitotoxicity by aggravating oxidative stress and inflammation, and inflammatory mediators might regulate extracellular glutamate levels by reducing the glutamate scavenging ability of glial cells, including microglia and astrocytes [7, 119].

Extensive activation of N-methyl-D-aspartate (NMDA), a glutamate-gated ion channel, is one of the major causes of glutamate toxicity. NMDA activation induces glutamate toxicity by suppressing the synthesis and release of neurotrophic factors, such as BDNF and other synaptic proteins [119]. It was shown that another α-amino-3-hydroxy-5-methyl-4-isoxazolepropionic acid (AMPA) glutamate receptor (GluR) increases the release of inflammatory cytokines [120], which, conversely, increases synaptic glutamate and potentially contributes to excitotoxicity, followed by a succession of events, including synaptic plasticity and memory impairment [121]. Simply put, inappropriate activation of GluRs eventually destroys the delicate balance of neuroprotective versus neurotoxic effects of glutamate in brain, substantially increasing the likelihood of mental disorders, such as depression. In support of this idea, many studies have reported that higher levels of glutamate and its metabolite were measured in the blood and CSF of patients with PSD, particularly in the frontal cortex [122, 123]. Also, the alteration of glutamate levels in the epileptic brain is well established. A microdialysis study by Çavuş et al. showed that, compared to the non-epileptogenic cortex, glutamate levels were elevated in epileptogenic, non-localized and lesional cortical sites [124]. With the increasingly clear role of glutamate in post-stroke damage, glutamate excitotoxicity might be a potential therapeutic target for these neuropsychiatric complications.

### 4.6. Mitochondrial dysfunction

The importance of mitochondrial dysfunction in the development of stroke-related brain damage is widely recognized [125]. Stroke induces depolarization of mitochondrial membrane potential (MMP), which initiates a succession of disastrous events, including aberrant reactive oxygen species (ROS) generation, reduced mitochondrial adenosine triphosphate (ATP) production, and Ca^2+^ accumulation, which exacerbates the dysregulation of mitochondrial biogenesis [126]. Deficiency of energy supply and metabolism is the most immediate cause of mitochondrial dysfunction in cerebral ischemia [127]. Furthermore, mitochondrial dysfunction-induced excess ROS leads to oxidative stress and lipid peroxidation, which activates microglia and, subsequently leads to neuroinflammation [128, 129]. Moreover, Ca^2+^ overload and the irreversible collapse of MMP potentially activate the mitochondrial permeability transition pore (MPTP) opening, followed by cytochrome C releasing from mitochondria into the cytoplasm, which, in turn, initiates the apoptotic cascade and results neuronal death [130]. Thus, these cascades induced by mitochondrial dysfunction affect complications of stroke involving dementia, epilepsy, and psychiatric disorder. The crucial role of mitochondrial dysfunction in post-stroke sequelae is further supported by the finding of impaired energy metabolism in patients with depression [130], as well as by evidence that enhanced cerebral ATP availability may have antidepressant effects [131]. Previous data also showed that ameliorating oxidative stress and mitochondrial dysfunction can promote the recovery of learning and memory function [132, 133], and transplantation of exogenous mitochondria exert the function of rescue neurons caused by ischemia damage [134].

## 5. Therapeutic intervention

### 5.1. Pharmacological treatment

Currently, multiple rehabilitative modalities are used to intervene in post-stroke complications, including pharmacological agents (Western and traditional Chinese medicine [TCM]), and non-pharmacological treatments such as acupuncture, TMS, and psychological counseling. Here, we discuss the mechanisms and effects of these therapies and explore more efficient or combined therapeutic strategies. The general characteristics of various treatments for post-stroke complications are shown in Table 1.

**Table 1:** The general characteristics of various treatments for post-stroke neuropsychiatric complications

Types of PSNCs	Study ID	Inclusion criteria	Treatment	Dose range	Type or mechanism of drug	Outcomes or conclusion	Adverse effects
Cognitive impairment and Dementia	Narasimhalu et al [165]	Ischemic stroke patients aged 55-85 years, with CI no dementia	Rivastigmine	1.5-4.5 mg twice a day.	Cholinesterase inhibitors	Well tolerated, potentially improve executive functioning.	Nausea, sleeping difficulties, headache, gastrointestinal upse, chest pain, giddiness, etc
	Dichgans et al [166]	Men and women aged 25-70 years, with CADASIL, meet the CI criteria: (1) a description of cognitive problems given by others. (2)a MMSE score of 10-27, or a TMT B time score 1.5 SDs below the mean	Donepezil	5 mg daily for the first 6 weeks and 10 mg daily thereafter	Cholinesterase inhibitor	Had no effect on the primary endpoint of the V-ADAS-cog score, had some improvements on executive function.	Gastrointestinal disorders, nausea, diarrhoea and vomiting, muscle cramps, dizziness, insomnia
	Kos et al [58]	Stroke in the right hemisphere with cognitive impairment	Donepezil	5 mg/day	Increased recruitment of the parieto-frontal networks	Significant improvements in the Mini-Mental Status Examination	/
	Jorge et al [113]	Patients aged 50-90 years, within 3 months following stroke	Escitalopram	10 mg/day for patients <65 years and 5 mg/day for patients ≥65 years	Selective serotonin reuptake inhibitor	Significant improvement of RBANS score	Dry mouth, constipation, indigestion, anorexia, etc
	Black et al [167]	Patients mean aged 73.9 years; with probable or possible VaD, according to criteria of NINDS and AIREN	Donepezil	5-10 mg/day	Cholinesterase inhibitor	Significant improvement in cognition, global function and activities of daily living	Nausea, diarrhea, cramps and leg cramps, anorexia, vomiting, headache, abnormal dreams, hypertension, etc
	Erkinjuntti et al[168]	Patients met the clinical criteria of probable vascular dementia	Galantamine	24 mg/day	Inhibits acetylcholinesterase and modulates nicotinic receptors	Great efficacy on ADAS-cog and CIBIC-plus, activities of daily living and behavioural symptoms	Nausea, vomiting
	Whyte et al [169]	Stroke within the last 30 days, and cognitive impairment	(1)Galantamine; (2)donepezil	(1)8-24 mg/day; (2)5-10 mg/day	Acetylcholinesterase inhibitors	Donepezil had better functional recovery than galantamine.	Gastrointestinal side effects
Pain	Vick et al [170]	A 68 years old female with central pain syndrome following an ICH	Ketamine	50 mg/night to 50 mg three times/day	NMDA antagonist	Beneficial in decreasing allodynia and hyperalgesia, improving functional capabilities	No side effect
	Lampl et al [171]	Patients aged 36 to 68 years, with central post-stroke pain	Amitriptyline	10-75 mg in extended-release form within 3 weeks then kept for 365 days	Elevate levels of serotonin and noradrenaline, NMDA receptor blockers	Reduces but does not completely prevent CPSP	No new side effect
	Kruszewski et al [172]	Patients aged over 18 years, with central neuropathic pain associated with spinal cord injury	Pregabalin	150-600 mg/day	Reduced calcium influx into hyperexcited neurons	Efficacious in relieving central neuropathic pain, improving sleep, anxiety, and overall status	Somnolence and dizziness
	Vestergaard et al [173]	Patients range 37 to 77 years, with CPSP	Lamotrigine	25-200 mg/day	Anti-glutamatergic and block sodium channel	A well tolerated and moderately effective treatment for CPSP	Mild rashes
	Pellicane et al [174]	Patients with CPSP	Methylprednisolone	4-12 mg/day	Not mentioned	Nearly equivalent effects versus other drugs (pregabalin, amitriptyline, lamotrigine)	/
	Hesami et al [175]	Patients with stroke in the thalamic area and diagnosed with CPSP	Gabapentin	300 mg twice daily for one month	Probably elevates GABA levels in the brain	Safe, efficacious, well tolerated and lack of interaction with other drugs	No adverse effects
	Shimodozono et al [112]	Patients with hemiplegia and intractable CPSP	Fluvoxamine	25-125 mg/day	Selective serotonin reuptake inhibitor	Useful for the control of CPSP in the early stage	Headache, itching of skin, appetite loss
	Jungehulsing et al [176]	Patients, age > 18 years, CPSP persisted > 3 months, and a pain score ⩾4 on the 11-point Likert scale	Levetiracetam	1000 mg BID to 3000 mg/day	/	Not effective in treatment for CPSP	Tiredness, pain increase, dizziness, pruritus and headache
Epilepsy	Gilad et al [138]	Patients with a first single epileptic seizure after an ischemic stroke	(1)Lamotrigine (LTG) (2)Carbamazepine (CBZ)	(1)25-100 mg every week (2)100-300 mg/day	(1)Inhibition of the voltage-dependent sodium channel and the release of glutamate. (2)/	LTG is a relatively better-tolerated drug than CBZ, and can be acceptable as initial treatment in post-stroke seizures	(1)Somnolence and dizziness (2)Somnolence, dizziness, nausea and vomiting, skin eruption, confusion and overdose symptoms
	Belcastro et al [177]	Patients, aged 71.9±7.3 years, with late-onset post-stroke seizures	Levetiracetam (LEV)	1000-2000 mg/day	/	A safe and effective candidate as a first-choice drug against post-stroke seizures.	Drowsiness, aggressive behaviour
	Kutlu et al [178]	Patients aged 60 or older with at least two late-onset post-stroke seizures.	Levetiracetam	1000-3000 mg/day	/	An efficacious and well tolerated therapy in elderly patients with late-onset post-stroke seizures	Somnolence, headache, dizziness
	Hsieh et al [153]	Human; hospitalized for the first-ever stroke	Statins include atorvastatin, rosuvastatin, fluvastatin, simvastatinand lovastatin	Not mentioned in detail	Possibly decrease isoprenoids of the β-Hydroxy β-methylglutaryl-CoA (HMG-CoA) reductase pathway	A modest drug with non-significant effect in preventing post-stroke epilepsy, decreasing post-stroke mortality only in men.	/
	Matsubara et al [140]	patients with a first-ever ischemic stroke and no history of epilepsy before stroke	Statins included pitavastatin, atorvastatin, simvastatin, rosuvastatin and pravastatin.	Not mentioned in detail	Probably inhibit HMG-CoA reductase and its neuroprotective properties	Reduction the risk of post-stroke early onset seizures, preventing the progression of initial post-stroke seizure-induced neurodegeneration into chronic epilepsy.	/
	Gilad et al [179]	Patients with spontaneous non-traumatic and non-aneurysmatic ICH	Valproic acid	800 mg/day	/	Improve neurological outcome and reduce early seizures, but did not prevent the occurrence of seizures post ICH	Mild liver dysfunction
	Alvarez-Sabín et al	Patients aged 18 years or older, with a first post-stroke late epileptic [180] seizures	Gabapentin	900-1800 mg/day	/	Useful and safe for late post-stroke epileptic seizures	Drowsiness and dizziness, headache, fatigue, nausea or vomiting, dyspepsia, weight increase and peripheral edema
Depression	Choi-kwon et al [152]	Patients with post-stroke depression, emotional incontinence, or anger proneness	Fluoxetine	20 mg/day	Selective serotonin reuptake inhibitor	Efficacious in the treatment of PSEI and PSAP; effect on PSD is not solidly confirmed.	Nausea, headache, insomnia, sexual dysfunction, etc
	Kim et al [181]	Older than 20 years, with an acute stroke within the past 21 days, had a modified Rankin Scale score of two or greater	Escitalopram	10 mg/day for 3 months	Selective serotonin reuptake inhibitor	Did not significantly reduce moderate or severe depressive symptoms in patients with acute stroke.	Constipation, muscle pain, insomnia, diarrhoea
	Ribeiro et al [182]	An 82-year-old man with no psychiatric history, severely depressed after stroke	(1)Sertraline (2)quetiapine	(1) 100 mg/day (2) 50 mg/day	Acting on frontal-subcortical circuits and related regions.	Full remission of depressed symptoms	Not mentioned
	Ding et al [151]	Aged 18-75 years; not taken antidepressants in the last 2weeks; meet the diagnostic criteria of stroke and depression, and HAMD>18 points;	(1)Danzhi Xiaoyao Powder (2)escitalopram oxalate	(1)3 bag/day (2) 10 mg/day	Probably inhibit hyperactivity of HPA and regulate neurotransmitters in hippocampus	/	Without increasing adverse effects
	Palomäki et al [183]	Ischemic stroke occurred not more than 30 days earlier, under 71years old	Mianserin	10-60 mg/day	Tetracyclic antidepressant Drug	Unable to prevent PSD	Diarrhea and nausea
	Rasmussen et al [184]	Diagnosed with stroke without current depression, significant aphasia or dementia, history of schizophrenia, psychosis, or severe drug abuse and antidepressants treatment	Sertraline	50-100 mg/day	Selective serotonin reuptake inhibitor	Have significantly superior prophylactic efficacy	Tiredness, weight gain, dry mouth, cheek smarting, and rash
	Robinson et al [185]	Nondepressed patients within 3 months following acute stroke	Escitalopram	10 mg/day < age 65, 5 mg/day > age 65	/	Significantly lower incidence of depression	Gastrointestinal adverse effects such as dry mouth, constipation, indigestion, etc; Sexual adverse effects such as decreased libido; Cardiovascular adverse effects such as tachycardia; Other adverse effects such as dizziness, fatigue, etc
Apathy	Tay et al [186]	Patients ⩾18 years between 2 and 15 days after stroke onset, with persisting neurological deficit	Fluoxetine	20 mg/day	Selective serotonin reuptake inhibitor	Ineffective in preventing post-stroke apathy	/
	Kohno et al [187]	An 80-year-old right-handed man with severe apathy after cerebral infarction in the prefrontal cortex	Ropinirole	0.75 mg/day	Selective agonist of dopamine D2/3 receptors	Remarkably improved verbal output and spontaneity in daily life	/
	Aragona et al [188]	A 62-year-old man affected by hemorrhagic stroke in the left thalamus, presented with marked apathy	Bupropion	150 mg/day	Improve the mesocortical pathway	Have an overall reduction of apathy	/
	Auret et al [189]	A 44-year-old patient with severe and disabling apathy nearly 2 years after a right hemisphere hemorrhagic stroke	Zolpidem	A single-dose of 10 mg/day on days 2, 4 and 5 over a 9-day period	/	Dramaticly improved apathy	No adverse effects
	Robinson et al [190]	Diagnosed with apathy	Nefiracetam	600 or 900 mg/day	/	900 mg nefiracetam had a significantly greater change in Apathy Scale scores	Gastrointestinal, nervous system side effects, etc
	Mikami et al [191]	Patients 50-90 years old within 3 months of an index stroke who did not meet DSM-IV diagnostic criteria for major or minor depression and did not have a serious comorbid physical illness	Escitalopram	10 mg patients ≤ 65 years; 5 mg patients ≥ 65 years	/	Effective in preventing new onset of apathy following stroke	Constipation, indigestion, anorexia, anorgasmia, ejaculation disorders, chest pain and tachycardia
	Watanabe et al [192]	A 38-year-old right-handed man with prominent apathy after subcortical infarcts	Methylphenidate	5mg bid	/	Selective improvement of frontal system function	/
	Starkstein et al [193]	Age 40 to 90 years, apathy onset 8 weeks after stroke	Nefiracetam	900 mg bid	/	Did not prove to be efficacious in post-stroke apathy	Fatigue, muscle or joint pain, etc. It did not produce more adverse events than placebo
Anxiety	Rao et al [194]	Diagnosed as post-stroke anxiety	Sertraline	25-200 mg/day	Selective serotonin reuptake inhibitor	Well tolerant, had improvement on the Clinical Anxiety Scale	No adverse events
	Mikami et al [191]	Aged 50 to 90 years old, an index stroke within 3 months and did not meet DSM-IV diagnostic criteria for depressive disorder	Escitalopram	10 mg/day ≤ age 65, 5 mg/day ≥ age 65	/	Effective in preventing new onset of post-stroke generalized anxiety disorder	No significant differences among different group
Fatigue	Choi-Kwon et al [152]	Patients with PSF	Fluoxetine	20 mg/day for 3 months	Selective serotonin reuptake inhibitor	Not effective in improving PSF	Not mentioned

Abbreviations: CI, cognitive impairment; CADASIL, subcortical infarcts and leucoencephalopathy; MMSE, mini-mental state examination; TMT, trail making test; V-ADAS-cog, vascular AD assessment scale cognitive subscale; RBANS, Assessment of Neuropsychological Status; VaD, vascular dementia; NINDS, National In-stitute of Neurological Disorders and Stroke; AIREN, the Association International pour la Recherche et l’Enseignement en Neurosciences; ICH, intracerebral hemorrhage; NMDA, N-methyl D-aspartate; GABA, gamma-Aminobutyric acid; PSEI, post-stroke emotional incontinence; PSEI, post-stroke anger proneness; HAMD; Hamilton Depression Scale; DSM-IV, Diagnostic and Statistical Manual of Mental Disorder

### 5.1.1. PSCID treatment

Thus far, there are no specific FDA approved pharmacological treatments for PSCID [16]. Several agents have been proved to be effective for vascular dementia, such as cholinesterase inhibitors (galantamine, donepezil, or rivastigmine), memantine and gingko biloba[135]. Clinical studies presented contradictory results on the effects of cholinesterase inhibitors. Stroke is a cerebrovascular accident, and PSCID is often the consequence of small cerebrovascular diseases. The treatment of PSCID should not be confined to anti-dementia medication, it may also benefit from therapy of anti-cerebrovascular disease [136].

### 5.1.2. PSE treatment

Current stroke guidelines do not recommend the administration of anti-epileptic drugs (ADs) for prophylactic treatment of PSE, owing to common adverse effects and drug-drug interactions, and evidence regarding use of ADs in PSE is scarce [137, 138]. A paucity of basic research and clinical trials investigating post-stroke has resulted in the situation that no valid medication is approved for the treatment of PSE. Further complicating the field, there is a lack of animal models that faithfully recapitulate post-stroke spontaneous epilepsy to enable robust preclinical studies. It is not surprising, however, that there is a divergence between evidence-based recommendations and clinical practice. Many clinicians prescribe ADs that are well-tolerated and lack significant drug interactions to prevent recurrent seizures when patients suffer an acute symptomatic seizure after stroke. Currently, clinical management of PSE is guided mainly by patient age, co-morbidities, and co-medications[139]. Over the last decades, several studies have shown that statins could decrease the incidence of epilepsy after stroke, although the results were inconsistent [137, 140].

### 5.1.3. CPSP treatment

A growing number of studies and trials have demonstrated the efficacy of pharmacological management in CPSP, including intravenous lidocaine and opioids as well as tricyclic antidepressants (TCAs), such as gabapentin, amitriptyline, and lamotrigine, which are recommended as first-line drugs [141]. In recent years, studies have found that aliphatic acid products and peptides may have potential in the treatment of CPSP by modulating the function of hippocampus [142, 143]. To date, the pharmacologic interventions available for CPSP are inadequate due to both drug resistance and side effects, particularly in elderly patients [143]. Furthermore, patients with CPSP often have co-morbidities, thus fully considering the necessity of prompt pain relief, side effects, and potential beneficial effects, as well as individualized medication selection is crucial to ensure appropriate management. Certainly, future drug development programs for CPSP should consider the high incidence of co-morbidities in this patient population.

### 5.1.4. PSD treatment

Pharmacological intervention is the main therapeutic approach to PSD, particularly in the sub-acute phase after stroke, and effective strategies are defined. Antidepressants used to treat PSD mainly include TCAs, SSRIs, dual serotonin and noradrenergic reuptake inhibitors, and these are the basis of current PSD intervention. Compelling clinical and preclinical evidence suggests that various phytochemical compounds extracted from natural products and compound prescriptions of TMC have strong antidepressant activities and few side effects, and these may represent promising new interventions for PSD [144-146]. Unlike clinically available agents, the mechanism of drugs being evaluated in basic research of PSD mainly focused on the regulation of the HPA axis and inflammation [147]. In addition, prophylactic use of antidepressants may be a feasible management strategy in stroke patients.

### 5.1.5. PSAp treatment

Available pharmacological treatments for PSAp are currently limited. Additionally, little basic research is being conducted on PSAp, and most published clinical studies are limited to anecdotal case reports and mainly focus on dopaminergic agents, cholinesterase inhibitors, and stimulants. Owing to the shared symptomatology with depression, many antidepressants, such as SSRIs, are prescribed for the treatment of apathy [148]. However, evidence showing that antidepressants have effects on patients without additional depressive symptoms is limited. More animal studies and controlled clinical trials are urgently needed to investigate the efficacy of current therapeutic options and to test other agents in PSAp.

### 5.1.6. PSAn treatment

Similar to the other post-stroke psychiatric disorders after stroke, no specific guidelines have been developed for the treatment of anxiety in stroke patients, and few clinical trials and no animal studies have specifically targeted PSAn symptoms. SSRIs, TCAs, benzodiazepines, and Z-drugs, such as zopiclone, zolpidem, and zaleplon, can, however, be used to treat general anxiety [149]. In addition, several studies have suggested that SSRIs might be effective for several anxiety disorders after stroke, and preliminarily evidence suggests that herbal medicine (HM) or HM combined with conventional pharmacotherapy may be a safer and more effective strategy for the treatment of PSAn when it is accompanied by other symptoms [150, 151].

### 5.1.7. PSF treatment

To date, there are no effective interventions for the treatment or prevention of PSF. Most current clinical practice guidelines rely on expert experience and consensus, which is partly attributed to the lack of understanding of its underlying mechanisms. An important consideration that has not been settled in the field is whether PSF actually requires medical therapy. Currently, pharmacological treatments for fatigue have been frequently investigated in patients with multiple sclerosis but rarely in patients with stroke. A considerable proportion of PSF management has focused on environmental suggestions and physical interventions. Even if depression and fatigue are generally dissociated, a few studies have reported relieving PSF with antidepressants, but these have achieved few definite effects [152]. Additional studies are required to clarify the causative factors of PSF and to explore effective treatments for PSF. Another noteworthy point is the application of statins: due to neuroprotective effects, statins are commonly prescribed to stroke patients, yet emerging studies have shown that statins result in a higher frequency of PSF, and part of the blame lied with their neuromuscular side effects [66]. It is necessary to raise awareness of this symptom among professionals and patients, and the use of statins should be vigilant when the evaluation of Fatigue Severity Scale (FSS) ≥4. Moreover, a retrospective cohort study has shown that statins only significantly reduce the long-term mortality of male patients after stroke [153]. Given the pro-PSF effect of statins and the truth that females predict long-term PSF more frequently, gender should be considered when prescribing statins.

### 5.2. Non-pharmacological treatment

#### 5.2.1. Acupuncture treatment

Acupuncture and moxibustion are prevalent in medicine, particularly in rehabilitation medicine, and show good efficacy and few adverse effects. In the last decade, there has been a marked increase in studies of acupuncture for intervention in PSNCs, most of which present positive outcomes. An overview of systematic reviews regarding acupuncture in post-stroke cognitive impairment and depression management concluded that acupuncture is safe and effective in improving cognitive function and depressive disorder without obvious serious side events for post-stroke patients [154]. Consistently, a systematic review and meta-analysis reported that acupuncture has a greater effect on PSD and a better safety profile than antidepressants [155]. Also, acupuncture can be an adjunctive treatment for post-stroke epilepsy, and patients who received acupuncture treatment experienced a decreased incidence of and a reduced risk of epilepsy [156]. Moreover, it has been shown that acupuncture is an effective treatment for post-stroke shoulder pain and central pain [157], and animal researches provide further support that electroacupuncture can effectively relieve CPSP by inhibiting autophagy in the hippocampus [93]. Nevertheless, the mechanism by which acupuncture affects the recurrence and rehabilitation of ischemic stroke remains poorly understood, and additional well-designed studies are required to elucidate its efficacy and mechanism.

#### 5.2.2. Other non-pharmacological treatment of PSNCs

Therapy for post-stroke complications is an unmet medical need [158]. Emerging technologies such as, transcranial magnetic stimulation (TMS), psychotherapy, behavioral therapy, educational programs and music or art therapy, have shown good efficacy in the treatment of post-stroke sequelae. Stroke patients often experience a strong psychological deterioration as a result of the sudden onset and potential loss of physical functioning, and psychosocial interventions are thought to be necessary for stroke survivors for whom monotherapy is ineffective, most notably those with psychiatric disorders. A randomized controlled trial revealed that a brief psychosocial-behavioral intervention plus pharmaco-therapy achieved better depression reduction and remission than antidepressant treatment alone in the short term and at a 2-years follow-up [159]. Strengthening the psychological expertise of healthcare professionals working on stroke units, and combining psychological counseling with nursing care, can effectively promote the rehabilitation of stroke patients. TMS has been shown to be beneficial for patients who are pharmaco-resistant. One randomized trial involving 13 patients with chronic stroke showed that high-frequency repetitive TMS over the dorsal anterior cingulate cortex to the medial prefrontal cortex ameliorated apathy compared to sham stimulation [160]. A meta-analysis by Shao et al. [161] showed that TMS could be an effective treatment for PSD and was more effective in Asians. In addition, using TMS to increase cortical excitability in patients with high levels of PSF resulted in improved fatigue symptoms that lasted for a week later. In recent years, music interventions have been used in post-stroke rehabilitation to contribute to the recovery of brain functions involved in movement, cognition, mood, and sensory perceptions. Several studies have indicated that music interventions have a positive impact on gait, extremity motor function, communication outcomes, and general well-being after stroke [162, 163]. To confirm these promising results, more high-quality studies including a larger number of patients are needed before specific recommendations can be made for clinical practice.

**Figure 3.**
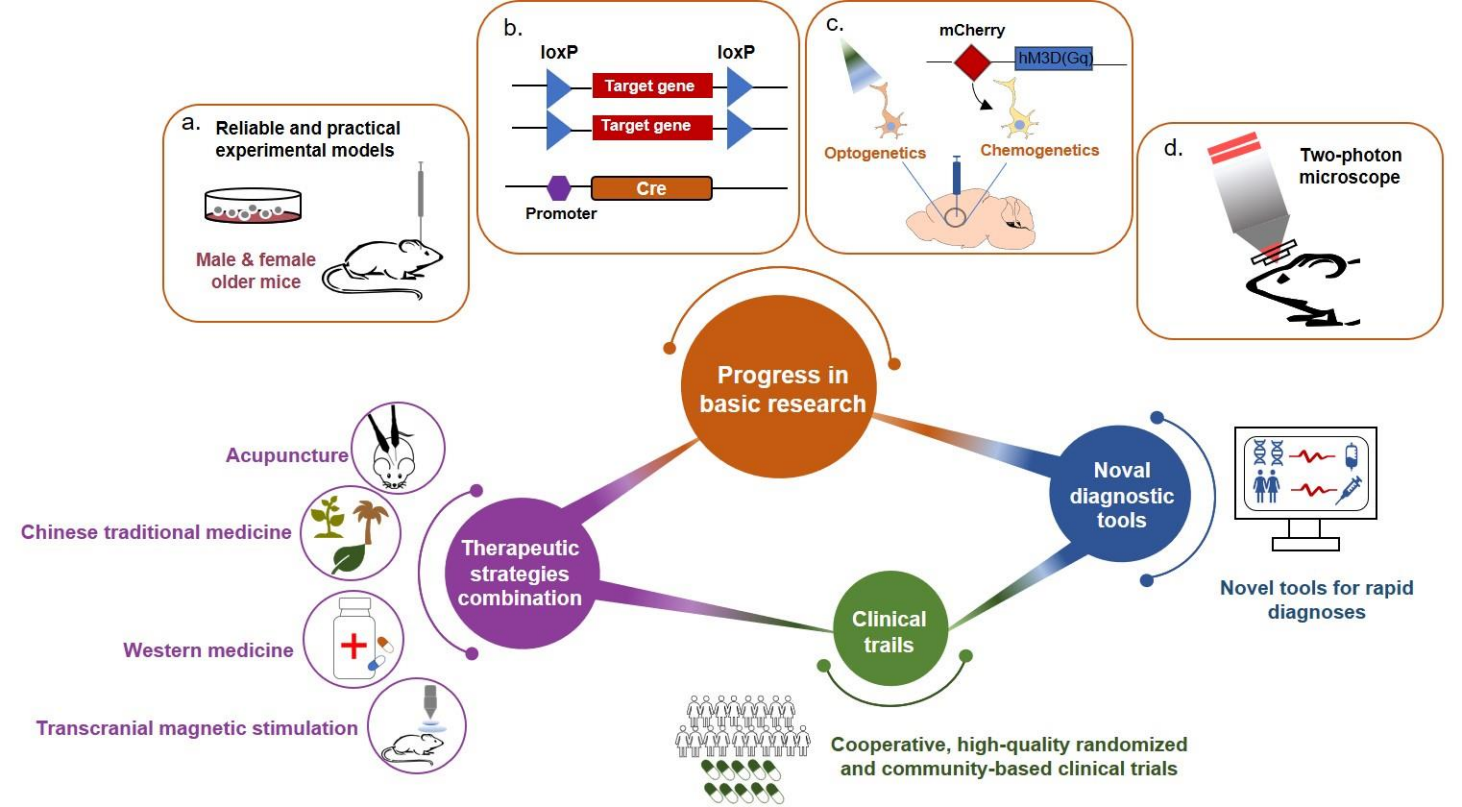
The perspective of future research in post-stroke neuropsychiatric complications. (1) Progress in basic research: a. Establish reliable and practical experimental models for PSNCs and b. Employ advanced pharmacological technologies. (2) Develop novel tools for rapid diagnoses. (3) Conduct cooperative, high-quality randomized and community-based clinical trials. (4) Combine multiple therapeutic strategies.

## 6. Conclusion and perspective

Although substantial progress has been made in the past two decades, the risk factors and the interplay of the underlying mechanisms mentioned above are insufficiently understood. More importantly, therapeutic interventions that are both effective and safe are lacking. Furthermore, the side effects of unsuitable treatments, probably resulting from the untimely diagnosis and complex clinical symptoms, should not be ignored. Taken all, further investigations into PSNCs, from both basic and clinical perspective, are urgent for the development of novel strategies for PSNCs (Fig. 3).

(1) Progress in basic research:
  a. Establish reliable and practical experimental models for PSNCs. So far, the basic research of mechanisms underlying PSNCs jog on slowly largely due to lack of repeatable animal models of PSNCs. Notably, existing laboratory models often use healthy young male animals, but stroke patients often have concomitant hypertension, diabetes, atherosclerosis, and other conditions. Many comorbidities must be taken into account when investigating novel animal models.
  b. Employ advanced pharmacological technologies. Currently, it is generally accepted that inflammation is involved in stroke-associated complications. However, how inflammation or inflammatory cytokines/ chemokines affect post-stroke phenotypes specifically remains unclear. Intriguingly, recent studies found that microglial synapse engulfment can be specifically promoted by IL-33 derived from astrocytes [164]. Therefore, it may be worth trying to dissect the cell-type-specific functions in the context of PSNCs by employing Cre-Loxp technology. In addition, many brain regions can provide cholinergic or serotoninergic effects to the cortex and hippocampus. However, it is still unclear which brain areas are responsible for cholinergic and serotoninergic dysfunction after stroke. The combination of optogenetics or chemogenetics with PSNCs animal models may be necessary to clarify this issue. Furthermore, in vivo two-photon imaging is an optional way to observe dynamic changes of immune cells after stroke; Detailed records of their movements (e.g., synaptic pruning or stripping by microglia) and morphology (e.g., microglial polarization) will help address the contradictory role of inflammation.
(2) Develop novel tools for rapid diagnoses. Stroke survivors often also have aphasia and limb incoordination, plus a strong social-psychological gap, which can make it difficult, or can make them reluctant, to articulate their needs and express their feelings. This can explain why some post-stroke symptoms are easily ignored by caregivers. Current instruments can already accurately locate the stroke lesions and provide high-definition images of the injured brain. These images can be re-used to establish the association of lesion patterns and post-stroke symptoms by a series of computational analyses, which will provide a valuable tool for predicting PSNCs.
(2) Conduct cooperative, high-quality randomized and community-based clinical trials. As mentioned above, most existing studies related to PSNCs have unignored limitations, including biased sampling, relatively small sample sizes and short follow-up periods, thus producing relatively unconvincing evidence. Hence, high-quality randomized clinical trials and community-based studies are urgently needed to fill existing current knowledge gaps.
(2) Combine multiple therapeutic strategies. Many approaches in addition to pharmaceutical interventions are also shown impressive protective effects against PSNCs. Therefore, the integrative applications of medicines and these treatment strategies, such as acupuncture and moxibustion, TMS, and environmental enrichment, are promising for the effective rehabilitation of stroke. However, standard clinical guidelines should also be proposed in time to avoid overtreatment.
